# Early multi-cancer detection using liquid biopsy: emerging biomarkers and clinical strategies

**DOI:** 10.3389/fonc.2026.1877911

**Published:** 2026-07-03

**Authors:** Sowhanur Rahman Nirob, Md Kishor Morol, Diya Rahman, Liew Tze Hui, Dip Nandi, Mashiour Rahman, Abdullah Al Jubair

**Affiliations:** 1 Department of Computer Science, American International University-Bangladesh (AIUB), Dhaka, Bangladesh; 2 ELITE Research Lab, Queens, NY, United States; 3 Faculty of Information Science and Technology, Multimedia University, Melaka, Malaysia

**Keywords:** biomarkers, CTCs, CtDNA, liquid biopsy, microRNA, multi-cancer early detection, NGS, proteomics

## Abstract

Liquid biopsy has become a revolutionary method for the early detection of cancer as a non-invasive technology that can assess circulating tumor material in biofluids. Liquid biopsy allows dynamic monitoring of tumor evolution, genetic changes and treatment responses, which is different from traditional tissue biopsy which offers a static and potentially narrow view of tumor biology. This mini-review will summarize the rapidly evolving future of circulating biomarkers (circulating tumor cells (CTCs), circulating tumor DNA (ctDNA), microRNAs, proteins, exosomes and epigenetic fingerprints), with their potential for early multi-cancer biomarkers, and their integration into early detection. The analytical sensitivity of liquid biopsy has expanded dramatically through technologies such as next-generation sequencing (NGS), digital PCR, and advanced proteomics. In addition, data collection has been enhanced through machine learning for increased predictive performance and the identification of new biomarkers. This review discusses clinical performance between liquid and tissue biopsy and the value of combined biomarker methods to improve accuracy for the detection of early disease. Finally, future directions will be presented to identify new methods of integration, improved costs and the establishment of early detection programs at the population scale.

## 1 Introduction

Detecting cancer early is a universally accepted factor in improving patient outcomes, survival, and quality of life. Although newly developed treatment regimens have greatly improved the prognosis of many patients, the most significant factor in the treatment’s success remains the stage at which the diagnosis is made. Standard approaches to diagnosis, through tests such as computed tomography (CT), magnetic resonance imaging (MRI), and ultrasound, provide anatomical and structural data; however, they often identify tumors after the disease is well developed and identifiable. In particular, they struggle to identify small lesions or even more subtle pre-molecular changes indicative of overt disease making patient diagnostic and treatment decisions more challenging. As a result, many cancers are only able to be identified and diagnosed after significant development and the clinical options for treatment may thus be more limited and the prognosis potentially less favorable Qureshi et al. (1)Feng et al. (2).

The usual or standard method of diagnosis is through a tissue biopsy, or surgical sample of tissue, which is still considered the gold standard. This method offers direct examination of tumor cells, giving detail molecular information about the tumor cells Zafar et al. (3)Qi et al. (4). However, this approach has its limitations, including, it may be invasive, and could cause unnecessary patient discomfort, bleeding or infection. Furthermore, tissue (biopsy) obtains only a small sample of the tumor, which may not be truly representative of heterogeneity of the tumor and misses potential metastatic tissue sites. As a result of these limitations it is impractical to subject a patient to multiple biopsies, and monitoring or tracking tumor growth or progression remains a challenge Bitin¸a-Barlote et al. (5).

To address these challenges, liquid biopsy has been recognized as an innovative and minimally invasive strategy. Liquid biopsy leverages the study of tumor-derived material in biofluids, most commonly blood, but also in urine, cerebrospinal fluid, saliva, reflects a dynamic, real-time understanding of cancer biology. CTDNA, CTCs, circulating tumor-derived extracellular vesicles (for example, exosomes), miRNAs, proteins, and epigenetic markers can and have been captured from these biofluids and all of these biomarkers capture, in aggregate, the genetic and phenotypic heterogeneity of tumors and may inform us about tumor evolution, dissemination potential and treatment response Imai et al. (6)Han et al. (7).

One of the most exciting applications of liquid biopsy is multi-cancer early detection (MCED). MCED seeks to detect multiple cancers from a single blood collection, potentially before clinical symptoms exist. Using the full power of molecular profiling, such as next generation sequencing, digital PCR or highthroughput proteomics, liquid biopsies can detect tiny amounts of tumor-derived material with exceptional sensitivity and specificity. Moreover, the ability to integrate and combine the information from multiple biomarkers is expected to provide a more comprehensive and accurate understanding of disease than can be achieved through single biomarkers Liang et al. (8)Sheriff et al. (9).

In addition to early detection, liquid biopsy provides important opportunities to monitor minimal residual disease (MRD), assess treatment effectiveness, determine the likelihood of relapse, and inform personalized therapy. For example, monitoring ctDNA levels after treatment with curative intent can identify residual disease as much as several months prior to recurrence identified via imaging, which allows intervention months earlier than was previously possible by conventional means Qi et al. (4)Neagu et al. (10)Connal et al. (11). In a similar fashion, CTC analysis can reveal information regarding tumor metastatic potential and aggressiveness that can inform clinical decision making about treatment Moon et al. (12).

The success of liquid biopsy widely depends on computational advances. Machine learning (ML) and artificial intelligence (AI) algorithms are now being used to allow researchers to analyze the integration of complex, high-dimensional (genomic, epigenomic, proteomic, and fragmentomic-based) data. These computational tools can significantly improve early cancer prediction, inform tissue of origin, and enhance risk-stratifying cancer treatment therapy, even in low tumor fractions where traditional assessments would have insufficient accuracy Liang et al. (8)Kulkarni et al. (13).

Yet progress does introduce challenges to consideration of liquid biopsy. The low availability of tumorderived material means that during early disease stages detection of samples may be present at volumetric levels that are generally not sustainable, confounding by non-cancer sources such as clonal hematopoiesis, and variability from sample collection, preparation, and storage incidents for each sample derived adds concerns to examination of any assay. Furthermore, the cost of sequencing their data combined with computational analyses yields further challenges. Standardized workflows, assays and computational pipelines must be developed if liquid biopsy is to transition to early bioscience stage of testing to clinical use practices Hao et al. (14)Liu et al. (15).

To conclude, liquid biopsy heralds a new era in oncology by providing non-invasive, serial, comprehensive detection, monitoring and management of cancer Qureshi et al. (1)Liu et al. (15)Di Capua et al. (16). By capturing a wide range of circulating biomarkers via recently developed analytical technologies and machine learning frameworks, liquid biopsy shows tremendous potential for population based multicancer early detection programs, individualized therapy, and enhanced patient outcomes. This review proposes to objectively navigate through the current biomarker landscape, the detection technologies, clinical uses, and their strategies to elucidate the future state of liquid biopsy for precision medicine.

## 2 Methodology of literature selections

**Figure 1:**
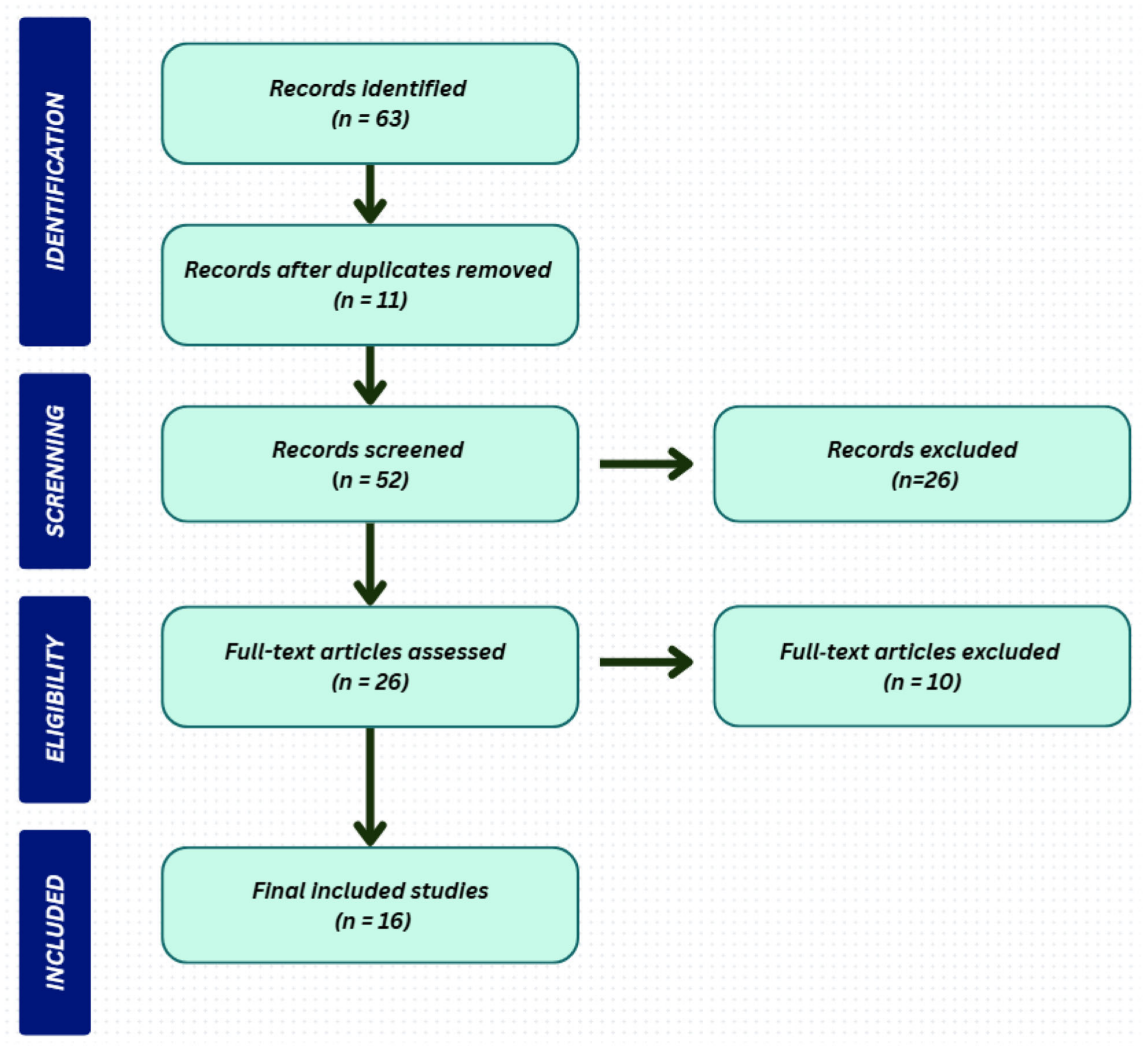
PRISMA 2020 flow diagram of study selection.

This mini-review employed a structured narrative literature search across primary scientific databases (PubMed, SpringerLink, PMC, ScienceDirect, and Wiley Online Library). The literature search window spanned from the years of 2021 to 2025, in order to capture the best evidence of advances in liquid biopsy technologies and multi-cancer early detection strategies. Search terms included “liquid biopsy”, “ctDNA”, “CTCs”, “early cancer detection”, “MCED”, “cancer biomarkers”, “microRNAs”, “NGS”, and “epigenetic signatures.” Articles were assessed for relevancy, rigor, and focus on identification of cancer earlier in the disease course as opposed to the management of advanced cancer. A PRISMA 2020-format flow diagram was added to transparently summarize the literature identification and screening process used in this narrative review. The study selection workflow is illustrated in **Figure 1**.

A total of approximately 63 initial articles were identified for potential inclusion, and sixteen articles were selected as representative sources that would exemplify great impact on the field of biomarker development and early detection for utilization in MCED Di Capua et al. (16) - Qureshi et al. (1). The publication’s literature provided scientific evidence that supports the positive contribution of circulating biomarkers, and technological applications that enable sensitive detection of early cancers.

## 3 Comparative analysis and discussion

### 3.1 Biomarker landscape in liquid biopsy

The principle of liquid biopsy is based on the idea that tumors shed molecular material into circulation. These circulating biomarkers serve as a non-invasive interface on tumor biology by reflecting genetic and phenotypic features. Biomarkers types in liquid biopsy currently focus on markers classified as circulating tumor cells (CTCs), circulating tumor DNA (ctDNA), microRNAs (miRNAs), proteins, extracellular vesicles, and epigenetic marks. Each type yields distinct but complementary insights to study early cancer detection or monitor disease.

To give an example of the scope of liquid biopsy biomarker types, **Table 1** shows a comparative overview of their biological nature, detection approaches, clinical and logistical benefits, and limitations.

**Table 1:** Comprehensive summary of biomarker classes used in liquid biopsy for early multi-cancer detection.

Biomarker type	Description	Detection principle	Key strengths	Major challenges
Circulating Tumor Cells (CTCs)	Intact malignant cells shed into the bloodstream from primary or metastatic tumors.	Isolated using immunoaffinity capture (EpCAM), microfluidic devices, or size-based filtration; analyzed through morphology, immunostaining, or singlecell sequencing.	Provides whole-cell information; enables analysis of metastasis dynamics and treatment resistance.	Extremely low abundance; high technical cost; inconsistent capture efficiency due to phenotypic heterogeneity.
Circulating Tumor DNA (ctDNA)	Short DNA fragments derived from apoptotic or necrotic tumor cells circulating in plasma.	Detected using digital PCR, targeted NGS, whole-genome and methylation sequencing.	Highly sensitive to early molecular changes; reflects tumor burden; useful for MRD detection and treatment monitoring.	Very low concentration in early Stage 0–I cancers; interference from clonal hematopoiesis; requires ultra-sensitive sequencing.
MicroRNAs (miRNAs)	Small non-coding RNAs regulating gene expression; released by tumor cells into blood or exosomes.	Quantified through qPCR, microarray profiling, or RNA-seq to detect dysregulated cancerspecific signatures.	Highly stable in biofluids; distinct cancer-type expression patterns; valuable for early-stage diagnosis.	Expression affected by inflammation or non-cancer conditions; non-standardized normalization methods.
Protein Biomarkers	Tumor-associated proteins, secreted factors, or altered circulating protein signatures.	Measured using ELISA, multiplex immunoassays, or proteomics (LC-MS).	Mature laboratory infrastructure; costeffective; historically used in screening (PSA, CA-125).	Low specificity; significant inter-patient variability; limited power for multicancer early detection.
Exosomes & Extracellular Vesicles	Nano-sized vesicles containing DNA, RNA, proteins, and metabolites released from tumor cells.	Isolated via ultracentrifugation, sizeexclusion chromatography, or immunocapture; molecular cargo profiled using sequencing or proteomics.	Cargo is biologically stable; carries multiomics information reflective of tumor microenvironment.	Isolation protocols lack standardization; signal may be diluted by vesicles from non-tumor sources.
Methylation Markers (cfDNA Methylome)	Cancer-specific epigenetic modifications detectable across circulating free DNA.	Profiling via bisulfite sequencing, NGS, or targeted methylation panels.	Highly tissue-specific; strong diagnostic power for MCED; robust even when ctDNA fraction is low.	Bisulfite conversion degrades DNA; high sequencing costs; computational complexity.
Fragmentomics	Patterns in cfDNA fragment size, end motifs, nucleosome footprints, and fragmentation profiles.	Genome-wide NGS fragment analysis and computational modeling.	Natural by-product of cfDNA release; useful when mutation burden is low; complements ctDNA sequencing.	Requires deep sequencing and advanced bioinformatics; interpretation still evolving; sensitive to pre-analytical variables.

### 3.2 Detection technologies for liquid biopsy biomarkers

**Figure 2:**
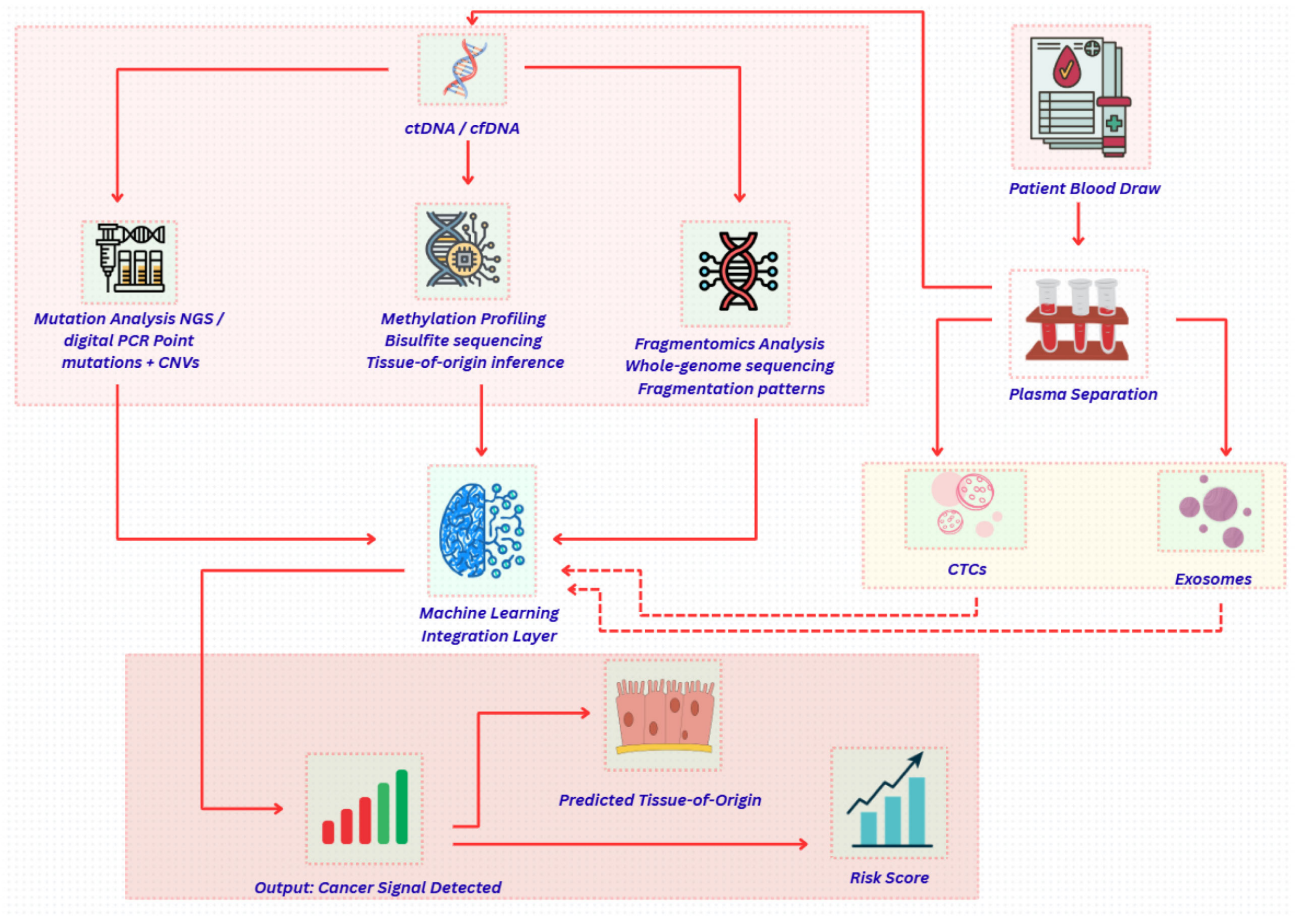
Schematic of the liquid biopsy MCED workflow.

The clinical usefulness of liquid biopsy is more dependent on the detecting technology of captured assays rather than the biomarker itself, bearing in mind the advantages and disadvantages of each platform and some controversies that are yet unresolved. For instance, assays based on PCR technology are still used because they are cheap, sensitive, and relatively uncomplicated to perform, but predetermined loci do curtail incidental mutations, epigenetic change, or broad changes in the genome that are unknown. There is still debate about the accuracy of these assays at very low variant allele frequency, but they must contend with biological noise and bias in certain instances with digital PCR (qualitative gains vs tradeoffs), although they can quantify better than traditional assays Bitin¸a-Barlote et al. (5). NGS has transformed the landscape of liquid biopsy for pathology by offering genome-wide evaluations of mutations, methylation, copy number alterations, and fragmentomic profiling for multi-cancer detection. There are many limitations to NGS-based tests, including cost, inter-lab variability, dependence on bioinformatic pipelines, and variability in sensitivity depending on TC, plasma volume or type, and source of tumor relationship to shed. The overall workflow of liquid biopsy-based multi-cancer early detection (MCED), including biomarker collection, molecular profiling and clinical interpretation is illustrated in **Figure 2**. Lastly, proteomics and multi-omic tests can provide further biological dimensions which may even have clinical value when nucleic acid has low signal, however introduction of these tests into clinical practice faces limitations testing for inherent pre-analytical variation in the form of standardized low and moderate, currently underdeveloped standardization and disclosure of expectations of value Imai et al. (6)Hao et al. (14). Finally, microfluidics and enrichment methods have been developed or designed for the isolation of circulating tumor cells (CTCs) and exosomes and provide a level of detail into the cellular and phenotypic characterization. These benefits come with challenges surrounding captures efficiency, reproducibility, and biological relevance. In the end, there is no one superior detection method, as the rigorous assay and clinical context will determine the best overall throughput of any detection method, and the only way to show real gains will be through head-to-head studies, validation with standardised workflows that are robust, and multicentre validations.

#### 3.2.1 Landmark MCED studies and platforms

Several landmark MCED studies have significantly advanced the clinical translation of liquid biopsy technologies for population-level cancer screening Qureshi et al. (1)Connal et al. (11). The CancerSEEK platform combined ctDNA mutation analysis with circulating protein biomarkers to improve multi-cancer detection and tissue-of-origin prediction, demonstrating the feasibility of multi-analyte bloodbased screening. CancerSEEK achieved a median sensitivity of approximately 70% across eight cancer types at greater than 99% specificity. Similarly, the Circulating Cell-free Genome Atlas (CCGA) study established the clinical utility of cfDNA methylation profiling with specificity exceeding 99% across diverse cancer types, although sensitivity for Stage I cancers remained relatively limited Imai et al. (6). The PATHFINDER study further evaluated the real-world implementation of blood-based MCED screening and demonstrated high cancer signal-of-origin prediction accuracy while reducing unnecessary diagnostic procedures Liang et al. (8). In parallel, the DELFI fragmentomics approach introduced genome-wide.

cfDNA fragmentation profiling as a promising strategy for detecting early-stage cancers with high specificity Connal et al. (11). Furthermore, the Guardant SHIELD assay demonstrated strong performance for colorectal cancer detection using integrated methylation and mutation analysis. Collectively, these studies highlight the transition of liquid biopsy from exploratory biomarker discovery toward clinically applicable multi-omics screening frameworks integrating genomics, methylomics, fragmentomics, proteomics, and machine learning-driven analytical models Kulkarni et al. (13) Liu et al. (15). Representative performance metrics from these landmark MCED studies are summarized in **Table 2**.

**Table 2:** Representative performance metrics from landmark MCED and liquid biopsy studies.

Study/platform	Biomarker strategy	Sensitivity	Specificity	Tissue-of-origin sccuracy
CancerSEEK	ctDNA + proteins	∼70% (varies by cancer type)	>99%	∼83%
CCGA/Galleri	cfDNA methylation	∼51.5% overall	99.5%	∼88−93%
PATHFINDER	cfDNA methylation	∼29% stage I	99%	∼97%
DELFI	Fragmentomics	∼73%	∼98%	High discrimination capability
Guardant SHIELD	cfDNA colorectal assay	∼83% CRC sensitivity	∼90% specificity	CRC-focused

Different MCED approaches possess distinct biological and analytical strengths. Mutation-based ctDNA assays provide high specificity but often suffer from reduced sensitivity in low-shedding early-stage tumors. Methylation-based approaches generally demonstrate stronger tissue-of-origin prediction and broader pan-cancer applicability, whereas fragmentomics can capture cancer-associated chromatin architecture changes even when mutation burden is limited. Multi-omics integration strategies increasingly outperform single-analyte platforms by leveraging complementary biological signals, although they also increase computational complexity, assay costs and standardization challenges Qureshi et al. (1)Imai et al. (6) Connal et al. (11).

### 3.3 Clinical applications of liquid biopsy biomarkers

Liquid biopsy biomarkers have the ability to support a variety of clinical applications (future implications and things as such). Nevertheless, evidence regarding the robustness of these indications is limited and varies significantly to the specific indication. CTDNA methylation and ctDNA fragmentation assays are new technologies that can potentially identify malignancies prior to onset of symptoms and when employing these technologies for population screening; however, the sensitivity of these technologies in identifying stage I and low shedding tumors is inconsistent and therefore can only be implemented as a means for population screening after significant evidence for a decreased mortality rate is confirmed Qi et al. (4). Minimal residual disease (MRD) detection is more robust in identifying patients with persistent disease than conventional imaging; however, it is currently unknown whether earlier detection of disease results in improved survival or merely results in increased treatment. Liquid biopsy is effective for identifying mutations that arise due to previous therapies to develop an optimal therapeutic regimen for patients. The discordance between plasma and tissue, as well as interference by clonal hematopoiesis, presents challenges in determining reliable clinical values of liquid biopsy Han et al. (7)Liu et al. (15). Although both ctDNA and circulating tumor cell (CTC) elevation correlate with cancer aggressiveness, ctDNA and CTC analyses already demonstrate important clinical utility in advanced cancer monitoring and therapeutic resistance assessment. However, their direct role in guiding treatment decisions within population-level early detection and screening settings remains insufficiently validated. Overall, due to uncertainty regarding the results of liquid biopsy; the cost of liquid biopsy; and the inequitable access to liquid biopsy; these barriers are limiting the uptake of liquid biopsy in the oncology clinical setting Di Capua et al. (16).

### 3.4 Comparison of liquid biopsy and tissue biopsy in clinical performance

The clinical uses of tissue and liquid biopsies differ but are complimentary, allowing for the detection of many cancers at early stages. A tissue biopsy will continue to be the standard for the initial diagnosis of cancer and provides the histology, architecture of the cancer, grade and immune profile of the cancer Qureshi et al. (1). Because tissue biopsy is invasive and only represents a small part of the tumor, the clinical application of tissue biopsy is very limited. Liquid biopsies can collect ctDNA, CTCs and exosomal biomarkers from many different tumor sites, allowing for more extensive genomic data and increasing the ability to monitor real-time responses to treatments and resistance development Hao et al. (14). Liquid biopsies do have a few limitations, including low sensitivity for very early stages of disease; the unpredictability of CTC and exosomal shedding; and potential false-positive cases due to clonal hematopoiesis. However, due to the minimally invasive nature of liquid biopsies, they can be performed on a longitudinal basis. The current data indicate that combining both types of biopsies is the best option for diagnosis; however, there is ongoing research examining the possibility of using liquid biopsy alone as a diagnostic tool Liang et al. (8).

### 3.5 Integrating CTCs and ctDNA for enhanced diagnostic feasibility

The combination of CTCs and ctDNA increases diagnostic feasibility as the two analytes will yield complementary, biological information that neither will provide alone. CTCs maintain intact cellular architecture, allowing for morphological analysis, protein biomarker profiling and insight into epithelial-tomesenchymal transitions, while ctDNA provides genomic and epigenomic information in high-resolution, with straightforward detection of mutations, methylation alterations and copy-number alterations with an acceptable sensitivity Liang et al. (8). Using combined assays improves detection rates overall. In any cancer, especially those cancers from either analyte alone would be inadequate. Combined assays also reduce the rate of false negatives in early-stage or low-shedding tumors by collectively sampling multiple routes of tumor spread. However, combining assays enables certain challenges. First, the ease of capturing a circulating tumor cell (CTC) is method dependent Liu et al. (15). Second, ctDNA assays differ by platform. Lastly, using multi-modal workflows can increase both the cost of samples and analytical complexity. It is important to note that even when combined detection generally improves analytic performance, there is no abundant evidence that multimodal strategies will improve patient outcomes. More prospective studies will be needed to determine the conditions under which and how CTC–ctDNA integration meaningfully supports diagnosis or prognosis and therapeutic decisions Imai et al. (6).

### 3.6 Machine learning and multi-omics integration in liquid biopsy

**Figure 3:**
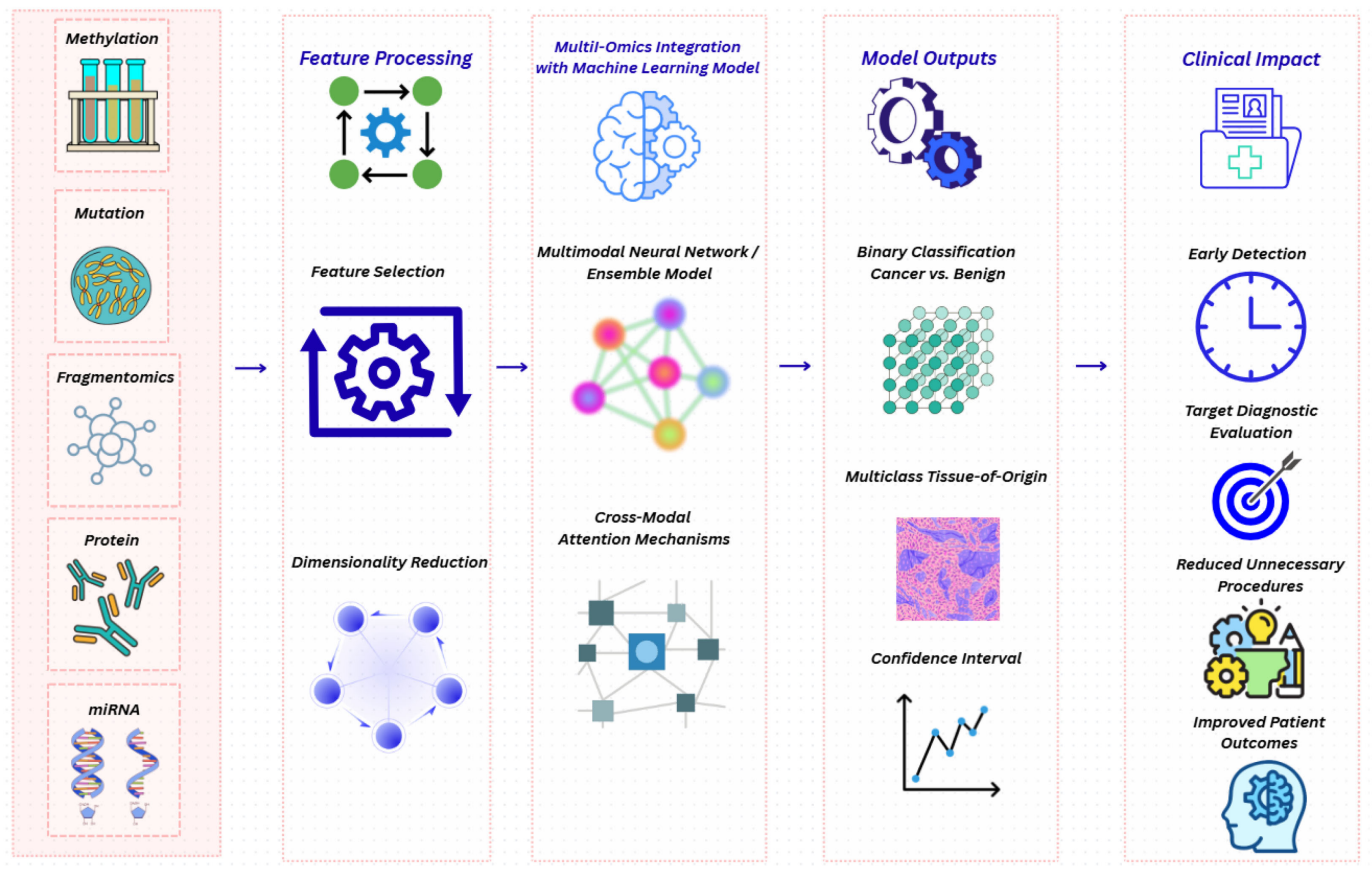
Multi-omics integration and machine learning framework for MCED.

Machine learning (ML) has become necessary for extracting clinically useful signals from highdimensional liquid biopsy data. This is principally true for multi-omic integration, as informative signals such as methylation, fragmentomics, mutations, and proteomic profiles must be collectively interpreted. ML also allows for sensitive and confident discrimination of malignant from non-malignant samples, makes organ-of-origin inference easier to interpret, and allows detection in low tumor fractions through pattern recognition that exceeds the human capacity to interpret Sheriff et al. (9). The integration of multi-omics biomarkers with machine learning algorithms for MCED is depicted in **Figure 3**. Nevertheless, assays based on machine learning (ML) encounter significant roadblocks like the risk of overfitting, performance decline across varied populations in the real-world setting, and lack of transparency in the pathway for models. External validation of ML across populations, laboratory processes, and sequencing modalities is scarce resulting in uncertainty about generalizability Kulkarni et al. (13). In addition, ethical and regulatory concerns including but not limited to data privacy, model drift, and lack of standardized reporting further complicate clinical uptake. As liquid biopsy-based assays become increasingly multi-omic, it will be imperative to develop transparent reproducible, and externally validated ML models to support clinical validity and regulatory acceptance. Recent advances in cancer AI increasingly employ multimodal deep learning architectures capable of integrating genomic, methylomic, fragmentomic, radiologic, and clinical datasets simultaneously. Federated learning has also emerged as a promising privacy-preserving strategy that enables collaborative model training across institutions without direct patient-level data sharing, potentially improving generalizability and reducing institutional bias in MCED model development Qi et al. (4).

### 3.7 Limitations and future direction of conventional diagnostics and liquid biopsy approaches

Challenges remain for conventional diagnostics in the clinical workflow and in liquid biopsy methods limiting their ability to perform adequately for clinical scalability. Tissue biopsy is required for histopathological confirmation but is an invasive procedure, subject to sampling bias, and not ideal for repeated monitoring. Liquid biopsy is minimally invasive (blood tests) and can provide a more complete picture of the variety of tumor types for any specific patient; however, these methods have shown inconsistent sensitivity for early-stage cancers due to low levels of tumor shedding and the specificity of these tests may be hindered due to various types of biological noise (clonal hematopoiesis). Additionally, there are multiple pre-analytic factors (collection, processing, storage) and high costs, combined with varying levels of healthcare capacity, that hinder the implementation of liquid biopsy. Pre-analytical variability remains a major challenge in liquid biopsy implementation. Blood collection tubes containing cell-stabilizing preservatives are preferred to minimize leukocyte lysis and contamination of circulating free DNA. Plasma separation is ideally performed within a few hours after collection to preserve analyte integrity, whereas long-term storage generally requires ultra-low-temperature conditions to reduce nucleic acid degradation. Another important challenge is clonal hematopoiesis of indeterminate potential (CHIP), which may introduce age-related somatic mutations unrelated to cancer and thereby generate false-positive ctDNA findings. Current mitigation strategies include paired leukocyte sequencing, advanced bioinformatic filtering, and integration of orthogonal biomarkers such as methylation and fragmentomic signatures. Future directions for liquid biopsy research will be focused on integrating multi-omics data, next-generation sequencing, AI-based biomarker discovery, standardized pipelines, and interoperable data frameworks to facilitate development of more accurate, cost-effective and accessible liquid biopsy platforms. However, large-scale trials will be required for demonstration of meaningful clinical impact.

## 4 Conclusion

Liquid biopsy has become a promising, low-risk method to obtain real-time molecular information that is representative of both primary tumor biology and metastatic biology. Liquid biopsy has distinct advantages to traditional diagnostics, including the capability for continuous surveillance over time, early detection of variants associated with therapeutic resistance, and improved diagnostics when sampling tissue is more difficult or unsafe. Yet, it is not fully integrated into standard clinical practice due to many outstanding challenges in integrating it into clinical diagnostic use, including reduced sensitivity to detect disease under the conditions of early malignancy, biological noise, in particular, clonal hematopoiesis, and variability across platforms and analytical pipelines. In addition to generating promising retrospective data, few prospective clinical trials have demonstrated the ability for liquid biopsy to directly impact survival or patient outcomes. The clear progress we hope to achieve in clinical practice will require establishing standardized workflows and analytical validation, using transparent computational methodologies, and performing large-scale studies to test the associations between liquid biopsy-guided patient decisions and improving patient outcomes. Liquid biopsy is poised to play a critical role as a component in precision oncology and through population-level cancer control with continued progress in multi-omics integration, decreasing costs, and creating harmonization across geographies.
